# Chrysophanic Acid in Psoriasis and Other Allied Skin Diseases

**Published:** 1880-01

**Authors:** A. F. Pattee


Chrysophanic Acid in Psoriasis and Other Allied Skin Diseases.—Chrysophanic acid is one of the most useful and important therapeutical agents we have in the treatment of certain intractable diseases of the skin, especially those belonging to the scaly order. I have treated many cases of psoriasis, herpes, eczema, impetigo, lichen and pityriasis, which have been of long duration, and have resisted other well-tried means, especially psoriasis. Three cases of more than ten years duration, whose bodies were almost entirely covered with scales, were cured after three weeks' treatment.

The acid can be used in connection with simple cerate, cod-liver oil, sweet oil, vaseline, etc.

℞.—Chrysophanic acid............ʒ ss. to ʒij

Vaseline....... ......................℥j

M. Sig.—Use on the diseased parts night and morning. Thirty grains of the acid is the smallest quantity used, and one hundred and twenty is the largest that will be required. In children suffering from scaly eczema, this ointment has been very successful. In three cases of pityriasis of the face of more than six years' duration, this ointment quickly cured. Herpetic and eczematous eruptions around the anus, prepuce and vulva that are stubborn to cure, rapidly improve after using this ointment and are soon cured.

In chronic ulcers of the leg from varicose veins or other causes, the following will prove an excellent application:

℞.—Ol. Terebinth, ℥j.

Acid Chrysophanic, ʒj.

M. Sig. Apply with camel's hair pencil, then cover the ulcer with the ointment. Renew every day for three days, then thickly apply oxide zinc ointment three times a day; use no soap or water about the limb, but if the ointment crusts on the leg remove it with sweet oil.—*A. F. Pattee, M.D., in Medical Brief.*

